# Specific Learning Disorders in Children and Adolescents with Obesity

**DOI:** 10.3390/children10101595

**Published:** 2023-09-24

**Authors:** Valeria Calcaterra, Laura Schneider, Stefano Baresi, Francesca Bodini, Federica Bona, Claudia Chillemi, Annalisa De Silvestri, Sara Zanelli, Gianvincenzo Zuccotti

**Affiliations:** 1 Department of Internal Medicine and Therapeutics, University of Pavia, 27100 Pavia, Italy; 2 Pediatric Department, Buzzi Children’s Hospital, 20154 Milano, Italy; laura.schneider@asst-fbf-sacco.it (L.S.); francesca.bodini@unimi.it (F.B.); federica.bona@unimi.it (F.B.); claudia.chillemi@unimi.it (C.C.); sara.zanelli01@universitadipavia.it (S.Z.); gianvincenzo.zuccotti@unimi.it (G.Z.); 3 Biometry & Clinical Epidemiology, Scientific Direction, Fondazione IRCCS Policlinico San Matteo, 27100 Pavia, Italy; a.desilvestri@smatteo.pv.it; 4 Department of Biomedical and Clinical Science, University of Milano, 20157 Milano, Italy

**Keywords:** specific learning disorders, children, adolescents, obesity, childhood obesity

## Abstract

Specific learning disorders (SLDs) are the most frequently diagnosed developmental disorders in childhood. Different neurocognitive patterns have been found in patients with overweight and obesity, but no data on childhood obesity and SLDs have been reported. To increase our understanding of the relationship between neuropsychological developmental and obesity, we assessed the prevalence of SLD in a pediatric population with obesity. We retrospectively included 380 children and adolescents with obesity. For all participants, auxological, metabolic, demographic features, relationship and social skills, anamnestic data on pregnancy and the perinatal period, stages of development and family medical history were reviewed. SLD was defined according to the DSM-5 criteria. A group of 101 controls of normal weight was included. The overall prevalence of SLD was 10.8%, and SLD was more prevalent in patients with obesity (*p* < 0.001), with male predominance (*p* = 0.01). SGA was associated with SLD (*p* = 0.02). Speech retardation (*p* < 0.001), limited relationships with peers (*p* < 0.001) and didactic support (*p* < 0.001) were noted in the SLD group compared to the group without SLD. A higher prevalence of family history of neuropsychiatric disorders was observed in the SLD group (*p* = 0.04). A higher fasting glucose level was detected in patients with obesity and SLD compared to subjects without SLD (*p* = 0.01). An association between obesity and SLD could not be excluded, and an overlap of pathogenic factors for both conditions should be considered.

## 1. Introduction

Obesity is the result of excessive body fat accumulation due to a positive energy balance and weight gain, due to the interaction of genes with the environment, lifestyle, and emotional and psychosocial factors [1,2,3].

In the countries of the European Region of the World Health Organization (WHO), obesity and overweight in childhood have been confirmed as one of the main public health problems, albeit with very different incidences across the various states [1].

Children suffering from obesity are more likely to maintain this condition into adulthood [2]; around 55% of obese children go on to have obesity in adolescence, and around 80% of adolescents will still have obesity in adulthood [3].

Obesity is associated with low-grade chronic systemic inflammation due to alterations of circulating cytokines levels and acute phase proteins [4]. The most important organ involved in the inflammatory process is the adipose tissue itself. The adipokines secreted are involved in an autocrine and paracrine pathway related to the regulation of energy expenditure, glucose and lipid metabolism, insulin resistance, endothelial function, and inflammation [4].

Obesity is a chronic multisystem disease related to increased morbidity and mortality [4] due to a strong association with different complications, including insulin resistance, type 2 diabetes, cardiovascular and respiratory disorders, chronic renal diseases, different types of malignant tumors, alterations in reproductive function, and neurodegenerative diseases [4,5,6,7,8,9,10]. Complication related to obesity usually seen in adults; however, there is literature evidence demonstrating that the initiating negative events related to obesity start early in childhood [4]. Obesity is a proinflammatory state that can affect nearly every organ in the body, and it has also been identified as a risk factor for the development of a wide variety of structural, physiological, psychological and behavioral disorders in both the central and peripheral nervous systems [6].

A link between obesity and specific neuropsychological developmental disorders, such as intellectual disability [11], autism spectrum disorder [12], depression [13] and anxiety [14], is recognized in pediatrics. An association between maternal prepregnancy overweight/obesity and a decrease in cognitive performance has also been reported, suggesting that an early pathogenic mechanism could not be excluded [15,16,17]. Although the exact mechanisms or mediators that underlie the connections between obesity and the risk of cognitive impairment remain unknown, an overlap of genetic, epigenetic and environmental factors has been proposed [18,19].

Numerous studies have shown that obesity and metabolism-related disorders are also associated with poorer cognitive performance and cognitive decline in adults [19,20]. As reported by Nguyen et al., subjects with morbid obesity also exhibit some cognitive weaknesses with regard to executive function, memory, processing speed and attention/vigilance, and these cognitive deficits resemble specific learning disorders (SLD) [19,20]. However, the literature does not provide conclusive data on the correlations between obesity and SLD.

SLD is a condition characterized by educational skills significantly lower than expected according to age that affect success and daily activities related to school or professional life [20,21]. Symptoms of SLD change through different age periods and are usually detected during early school age [22]. SLD is the most frequently diagnosed developmental disorder in childhood. The prevalence rates of SLD vary according to age, sex and developmental process, including puberty [23]. Even though the pathogenesis of SLD is not known, several conditions have been described as risk factor for SLD, such as family history of SLD, understimulating environments, prematurity, developmental and mental health conditions, neurologic conditions, history of brain injury, chromosomal disorders [22,23,24,25,26]. As reported by McBride [27,28], types of culture, language and script are relevant factors in reading and learning difficulties; despite bilingualism itself not being a risk factor for SLD, it may be associated with reading difficulties in some subjects [27,28,29].

Data on the prevalence of SLD in under- and over-nourished eating disorders (ED) during childhood and adolescence are limited. In 2022, for the first time in Italy, Aruta et al. [30] assessed the prevalence of SLD in a population of 262 patients affected by EDs and found a statistically significant increase of 9.54%. SLD diagnosis was not associated with a higher frequency of ED diagnosis or with psychiatric comorbidity in general [30]. EDs, such as binge eating disorder (BED), are frequently associated with overweight or obesity [30]. As recently reported, obesity and ED are intimately connected and could be considered as part of a continuum, sharing pathogenic mechanism and the same repercussions in term of psychosocial, metabolic and nutritional health [31,32,33]. Different neurocognitive patterns related to the presence/absence of BED have been found in patients with overweight/obesity [11,12,13,14,15,16,17]. No data on childhood obesity and SLD have been reported.

In this preliminary study, we aim to assess the prevalence of SLD in a population of children and adolescents suffering from obesity. SLD and obesity are considered to be disorders with a biological origin in which the interaction of genetic, epigenetic, and environmental factors has been described, and an overlap of pathogenic factors cannot be excluded. Increased understanding of the relationship between neuropsychological developmental and obesity would be useful for optimizing care and assisting this affected pediatric population.

## 2. Patients and Methods

### 2.1. Patients

We retrospectively included 380 children and adolescents (176 females and 204 males) with obesity (BMI-z score ≥ 2 according to the World Health Organization, WHO), aged 6–15 years who had been referred to the outpatients’ clinic of the Buzzi Children’s Hospital, Milano, Italy for obesity by their general practitioner or primary care pediatrician between January 2021 and May 2023. Known secondary obesity conditions, concomitant chronic illnesses and neurodevelopmental disorders were considered exclusion criteria.

We also evaluated 101 healthy children and adolescents of normal weight who were enrolled as controls. All parents gave their consent to participate retrospectively in other studies for clinical research purposes, epidemiology, study of pathologies, aimed at improving knowledge, care and prevention.

For all participants, data on previous SLD diagnoses were considered.

### 2.2. Methods and Procedures

#### 2.2.1. Specific Learning Disorders

SLD was defined taking the DSM-5 criteria [21] into consideration: subjects aged between 6 and 15 years, attending the second semester onwards of the first year of primary education, in absence of severe hearing and/or visual defects, not being diagnosed with chronic medical (epilepsy, cerebral palsy, encephalitis, etc.) or neurodevelopmental disorders (autism spectrum disorder, mental retardation, etc.) or defined syndrome or marked disturbance in speech.

#### 2.2.2. Procedures

For all patients, auxological and metabolic parameters were recorded at the first evaluation.

- Auxological evaluation

In all subjects, weight, height, BMI, waist circumference (WC), WC/height ratio and pubertal stages were considered. Systolic and diastolic pressure were also recorded.

Weight, height, WC, and blood pressure were evaluated as previously described [34,35]. In brief, height was measured with children standing barefoot with a Harpenden wall-mounted stadiometer (Holtain Ltd., Crosswell, UK) [34,35]. The weight was recorded with patients wearing underwear and standing upright in the center of the scale platform [34,35]. Waist circumference was measured in the horizontal plane midway between the lowest rib and the iliac crest using a flexible steel tape [34,35]. Systolic arterial pressure and diastolic arterial pressure were measured twice using a mercury sphygmomanometer, with an appropriately sized cuff on the right arm, after 5 min; the second measurement was used for analysis [34,35].

BMI was calculated as body weight (kg) divided by height squared (m^2^) and was transformed into BMI z-scores using WHO references [36].

The stage of puberty was classified in accordance with Marshall and Tanner [37,38], as follows: prepubertal stage = Tanner 1; middle puberty = Tanner 2–3; late puberty = Tanner 4–5.

- Biochemical evaluation

Blood was taken from subjects in a fasting state between 8:30 a.m. and 9:00 a.m., and plasma glucose, insulin, triglycerides (TG), total and high-density lipoprotein (HDL) cholesterol levels were measured using clinical chemistry methods via the Advia XPT (Siemens Healthcare). The homeostasis model assessment of insulin resistance (HOMA-IR) index [39] was used as a surrogate marker of insulin resistance (IR), and it was calculated as insulin resistance  =  (insulin × glucose)/22.5 [39].

Demographic features, relationship and social skills (bilingualism, relationships with peers, didactic support), anamnestic data on pregnancy and perinatal period (birth weight, gestational age, prematurity) and stages of development were also considered. Family medical histories were reviewed to identify neuropsychiatric disorders and SLD.

### 2.3. Data Analysis

To summarize the data, categorical variables are described as count and percentages and compared between groups with chi square test or Fisher exact test as appropriate. Quantitative variables are reported as mean and standard deviation (SD) if normally distributed (Shapiro–Wilks test), and as median and interquartile range (IQR) otherwise; they were compared between groups using the t-test for independent samples or the Mann–Whitney test, as appropriate. The analysis was performed using the Stata v17.0. program.

## 3. Results

Table 1 sets out the clinical and sociodemographic features of the patients and controls. No differences in age, sex or pubertal stage were noted between groups.

Patients with obesity showed higher birth weight, gestational age, weight, height and BMI, WC/Height ratio (*p* < 0.001); a significant difference in blood pressure level was also noted in patients with obesity compared to controls (*p* = 0.01).

Non-Caucasian ethnicity and bilingualism were more frequent in the obesity group (*p* ≤ 0.001). The prevalence of impaired relationships with peers and didactic support after introduction to elementary school was higher in patients with obesity than in the controls (*p* < 0.001).

The overall prevalence of SLD was 52/481 (10.8%), and it was more prevalent in patients with obesity (51/380, 13.4%) than in the controls (1/101, 1%) (*p* < 0.001).

In the patients with obesity, dyslexia, dysgraphia and dyscalculia were noted in 24/51 (47%), 9/51 (17.6%) and 7/51(13.7%), respectively, and more than one learning domain disorder was noted in 9/51 (17.6%). In one control, dyslexia and dyscalculia were reported.

Table 2 illustrates the clinical characteristics of the patients with obesity, with and without SLD.

SLD in patients with obesity was more predominant in males than in females (*p* = 0.01).

No significant differences in age at evaluation (*p* = 0.08), BMI (*p* = 0.14), pubertal stages (*p* = 0.32) or ethnicity (*p* = 0.30) were noted.

SGA occurred more frequently in patients with SLD compared to subjects without SLD (*p* = 0.02).

Speech retardation (*p* < 0.001), limited relationships with peers (*p* < 0.001) and didactic support after introduction to elementary school (*p* < 0.001) were all associated with SLD.

The prevalence of bilingualism was similar between groups.

A higher prevalence of a family medical history of neuropsychiatric disorders was reported in the SLD group (*p* = 0.04), with no difference in familiarity for SLD (*p* = 0.31).

As reported in Table 3, with the exception of a higher fasting glucose level in patients with obesity and SLD compared to the subjects without SLD (*p* = 0.01), no differences in metabolic parameters were noted between the groups.

## 4. Discussion

We observed a higher prevalence of SLD in a pediatric population suffering from obesity, compared to controls, with male predominance. An association between the presence of SLD and SGA, social interactions and a family medical history of neuropsychiatric disorders was detected. Patients with obesity and SLD also showed higher fasting blood glucose levels than the controls.

Learning disabilities are neurological conditions that affect the ability of the brain to send, receive, and process information [21,22]. In some cases, the learning difficulties are temporary and can be corrected with adapted interventions; for other children, learning skills of a mild enough level of severity can be compensated. However, for from 5% to 15% of children, these impairments are persistent [21,22]. The hereditary and genetic component is one of the underlying causes of learning disorders; however, the relationship between genes and the environment should be considered [21,22]. The overall prevalence of learning disorders, defined according to the DSM (including impairment in reading, writing and mathematics), is about 5% to 15% worldwide [21].

In our pediatric population, the overall prevalence of SLD was 10.8%, with higher percentages of cases in children with obesity (13.4%) compared to the controls (1%). In adults with learning disabilities, the prevalence of obesity is higher compared to that in the general population [40,41], and an association between obesity and SLD has been reported [19]. We are the first to provide data on its prevalence in a pediatric population with obesity, supporting a potential association also in pediatrics.

Obesity is a multifaceted disease with multisystemic involvement even at pediatric age [4,5,6,7,8,9,10,42,43] that increases the risk of the most common non-communicable chronic diseases, including metabolic, cardiovascular and respiratory diseases, cancer, and neurological disorders [4,5,6,7,8,9,10,42,43]. Many human brain diseases are associated with obesity, such as neurodegenerative conditions, neurodevelopmental disorders, and neuropsychological and psychiatric diseases [6,11,12,13,14,15,16,17,18,20].

The causal link between obesity and brain involvement has yet to be fully elucidated, but interaction between the peripheral metabolism and central brain function, inflammation, genetics, epigenetics, oxidative stress and bidirectional interactions between the gut microbiota and the nervous system in modulating neuroinflammatory signals has been proposed [6,11,12,13,14,44].

In this preliminary study, we observed that SLD was more prevalent in boys than girls [21,45], supporting the notion that sex differences in learning abilities and disabilities may exist [46]. Differences in learning styles, hormonal milieu, pre-perinatal complications, and maturation rate in boys compared to girls have been described as relevant factors to explain the male preponderance [46].

As reported by Gorked [43], SLD is a multifactorial disorder in which genetic predisposition, family load, cognitive and developmental factors, language spoken and bilingualism, and environmental factors may be relevant. Additional factors such as a family history of learning disabilities, premature labor, low birth weight and low Apgar have also been suggested as predeterminants. In our children, an association with prematurity was not detected. Conversely, an influence of birth weight was noted, confirming that many physical and psychological characteristics may be influenced by prenatal development and a link between low birth parameters and behavioral problems is not excluded [47]. In particular, according to the literature [47,48,49,50], children with an inappropriate weight for gestational age are vulnerable to experiencing learning difficulties. An association between SGA and SLD was confirmed in our population, supporting that birthweight may be an early developmental indicator of adverse school outcome [47,48,49,50].

Conversely, we noted an association with family medical history for neuropsychiatric disorders, but not with familiarity for SLD; additionally, no association with bilingualism was found. Patients with obesity showed impaired relationships with peers compared to the controls, and this association was more evident in patients with SLD; these data confirm that obesity may lead to lower social competence [51].

Considering the involvement of potential factors in the early years of life, such as gestational age, birth weight, Apgar score and maternal prepregnancy overweight/obesity [43], an early pathogenic mechanism of SLD is not excluded. According to the developmental origins of health and disease (DOHaD) theory, environmental factors influence offspring and also affect health and risk of disease in adulthood suggesting an interdependence of developmental influences, genes, and environment [52].

As reported by Buss et al., the fetal brain is highly plastic and requires cues from its environment to develop properly [53]. Brain development is a product of the bidirectional and dynamic interplay between the genotype of individuals and the nature of the early environment, from embryonic and fetal life to birth and infancy, and into childhood and adolescence [53]. A negative prenatal environment may represent a risk factor for the onset of neurodevelopmental disorders, including SLD [54].

Similarly, fetal programming involves the earliest stage of obesity development [52,53,55,56]. There is evidence that exposure to an abnormal in utero environment alters the metabolic programming of the growing fetus, increasing the lifelong risk of obesity.

Although the pathophysiological mechanisms of fetal programming are complicated and not well defined, epigenetic modification is a crucial factor. An overlap of epigenetic modifications causing both neurodevelopmental disorders and obesity cannot be excluded [18,57], and could suggest a future direction for expanding this field of research.

In our group of children with SLD, higher values of fasting blood glucose were recorded compared to the group without SLD. Diabetes has been reported to co-occur with developmental and learning disorders [58,59]. According to the 2016 Public Health England NHS Digital report, there are higher rates of both types of diabetes in all age groups in the SLD population compared to the general population, and these have an earlier onset, which is recorded at a younger age [60]. As reported by Cacciatore, glucose homeostasis may interfere with brain structure development and cognitivity, including learning anomalies [61]. These data may also support an overlap between behavioral and developmental disorders and glucose metabolism in young people.

We recognize that this study has some limitations, the first being its retrospective nature. Secondly, the number of participants was relatively small, and could limit the generalizability of the results. Finally, the SLD diagnosis was not performed in a unique specialized center. Despite the limitations, this preliminary study is likely to enrich the literature, providing useful information for further study.

In conclusion, we noted a higher prevalence of SLD in patients with obesity compared to subjects of normal weight, supporting a connection between neurocognitive processes and pediatric obesity. Sex difference, birthweight, and metabolic profile seem to be factors of consideration in patients with obesity and SLD. An association between obesity and SLD could not be excluded, and an overlap of early pathogenic factors for both conditions should be considered in pediatrics. An early identification of developmental markers, starting in the early period of life, is useful for providing timely interventions. Recognizing SLD in time is crucial for starting appropriate treatment and offering proper support to children and their families from an early age. Special attention and health surveillance could be provided to patients subjected to a negative environmental impact during the fetal period. Studying the link between alterations in the early life environment and increased susceptibility to obesity and neurodevelopmental disorders in depth could play a crucial role in the advancement of scientific knowledge of pathogenic disease mechanisms. Using longitudinal designs to evaluate associations between SLD and weight across different developmental periods may be useful for identifying which aspects of learning disorders are uniquely associated with adiposity indicators and when such associations emerge. Understanding the determinants of SLD is important in order to identify opportunities for personalized programs aiming at their prevention.

## Figures and Tables

**Table 1:** Characteristics of the patients with obesity and controls.

Features	Controls (*n* = 101)	Patients with Obesity (*n* = 380)	*p*
Age (years)	8.8 ± 3.8	9.0 ± 2.9	0.76
Sex (M/F)	34/67	204/176	0.87
Birth weight (g)	2974.9 ± 495.9	3298 ± 583.1	<0.001
Gestational age (weeks)	37.9 ± 2.4	39.2 ± 0.1	<0.001
Small for gestational age	6.9%	4.5%	0.32
Prematurity	9.2%	11.9	0.42
Weight (kg)	29.6 ± 12.5	54.5 ± 20.2	<0.001
Height (cm)	129.8 ± 25.5	142.4 ± 0.9	<0.001
BMI (kg/m2)	16.2 ± 2.0	25.9 ± 4.5	<0.001
Waist circumference/height ratio	0.46 ± 0.03	0.57 ± 0.06	<0.001
Systolic blood pressure (mmHg)	102 ± 8.7	106 ± 11	0.01
Diastolic blood pressure (mmHg)	63.5 ± 57.0	67.2 ± 10.0	0.01
Pubertal stage			
Prepubertal	63.0%	50.8%	0.03
Middle puberty	19.6%	34.7%	
Late puberty	17.4%	14.5%	
Specific learning disorders	1%	13.4%	<0.001
dyslexia	0%	47%	
dysgraphia	0%	17.6%	
dyscalculia	0%	13.7%	
more than one learning domain disorder	1%	6%	
Ethnicity			
Caucasian	88.1%	63.9%	<0.001
Others	11.9%	36.1%	
Bilingualism	16%	43.6%	<0.001
Speech retardation	5.94%	7.11%	0.68
Adoption	0%	0.5%	0.46
Relationships with peers			
Good	99%	87.4%	0.001
Impaired	1%	12.6%	
Didactic support	1%	15.5%	<0.001
Family medical for			
SLD	0%	0.08%	0.37 0.07
Neuropsychiatric disorders	0%	3%	

**Table 2:** Clinical characteristics of the children with obesity, with or without specific learning disorders (SLD).

Features	Patients with Obesity without SLD (*n* = 329)	Patients with Obesity and SLD (*n* = 51)	*p*
Age (years)	9.7 ± 3.0	10.4 ± 2.1	0.08
Sex (M/F)	46%/54%	53%/47%	0.01
Birth weight (g)	3303.0 ± 597.6	3217.3 ± 484.2	0.34
Gestational age (weeks)	39.3 ± 1.9	39.0 ± 1.9	0.35
Small for gestational age	4.6%	6%	0.02
Prematurity (yes)	8.2%	15.6%	0.08
Weight (kg)	53.6 ± 20.4	59.6 ± 18.9	0.04
Height (cm)	141.5 ± 18.2	147.7 ± 15.3	0.02
BMI (kg/m^2^)	25.8 ± 4.7	26.8 ± 4.4	0.14
Pubertal stage			
Prepubertal	52.5%	41.2%	0.32
Middle puberty	34.4%	35.3%	
Late puberty	13.1%	23.5%	
Ethnicity			
Caucasian	63.1%	70.6%	0.30
Others	36.9%	29.4%	
Bilingualism	44.9%	34.0%	0.14
Speech retardation	4.88%	19.6%	<0.001
Adoption	0.3%	1.9%	0.12
Relationships with peers			
Good	91.2%	64.7%	<0.001
Impaired	8.8%	34.3%	
Didactic support	9.1%	54.9%	<0.001
Family medical for			
SLD	0.6%	1.96%	0.31
Neuropsychiatric disorders	2.4%	7.84%	0.04

**Table 3:** Clinical features of the patients with obesity, with or without specific learning disorders (SLDs).

Features	Patients with Obesity without SLD	Patients with Obesity and SLD	*p*
Fasting glucose (mg/dL, nv <100)	86.1 ± 9.1	89.5 ± 1.0	0.01
HbA1c % (mmol/mol, nv < 42)	33.9 ± 3.7	33.6 ± 7.0	0.67
HOMA-IR	3.6 ± 3.4	4.0 ± 2.6	0.43
Total cholesterol (mg/dL, nv < 190)	163.6 ± 32.5	157.0 ± 34.0	0.21
HDL cholesterol (mg/dL, nv > 40)	48.0 ± 11.7	45.3 ± 11.5	0.16
Triglycerides (mg/dL, nv < 150)	90.0 ± 43.2	96.9 ± 44.3	0.32
Waist circumference/height ratio (vn < 0.5)	0.57 ± 0.08	0.58 ± 0.07	0.58
Systolic blood pressure (mmHg, vn < 120)	105.3 ± 10.8	105.5 ± 10.3	0.93
Diastolic blood pressure (mmHg, vn < 80)	66.6 ± 9.8	66.8 ± 9.3	0.89

HOMA-IR = homeostasis model assessment of insulin resistance; HDL = high-density lipoprotein; nv = normal value.

## Data Availability

Not applicable.

## References

[1] WHO Regional Office for Europe (2022). Report on the Fifth Round of Data Collection, 2018–2020: WHO European Childhood Obesity Surveillance Initiative (COSI). https://www.who.int/europe/publications/i/item/WHO-EURO-2022-6594-46360-67071.

[2] Llewellyn A., Simmonds M., Owen C.G., Woolacott N. (2016). Childhood obesity as a predictor of morbidity in adulthood: A systematic review and meta-analysis. Obes. Rev. Off. J. Int. Assoc. Study Obes..

[3] Simmonds M., Llewellyn A., Owen C.G., Woolacott N. (2016). Predicting adult obesity from childhood obesity: A systematic review and meta-analysis. Obes. Rev. Off. J. Int. Assoc. Study Obes..

[4] Calcaterra V., Regalbuto C., Porri D., Pelizzo G., Mazzon E., Vinci F., Zuccotti G., Fabiano V., Cena H. (2020). Inflammation in Obesity-Related Complications in Children: The Protective Effect of Diet and Its Potential Role as a Therapeutic Agent. Biomolecules.

[5] Kumar S., Kelly A.S. (2017). Review of Childhood Obesity: From Epidemiology, Etiology, and Comorbidities to Clinical Assessment and Treatment. Mayo Clin. Proc..

[6] O’Brien P.D., Hinder L.M., Callaghan B.C., Feldman E.L. (2017). Neurological consequences of obesity. Lancet. Neurol..

[7] Maggio C.A., Pi-Sunyer F.X. (2003). Obesity and type 2 diabetes. Endocrinol. Metab. Clin. N. Am..

[8] Jiang Z., Wang Y., Zhao X., Cui H., Han M., Ren X., Gang X., Wang G. (2023). Obesity and chronic kidney disease. Am. J. Physiol.. Endocrinol. Metab..

[9] Valenzuela P.L., Carrera-Bastos P., Castillo-García A., Lieberman D.E., Santos-Lozano A., Lucia A. (2023). Obesity and the risk of cardiometabolic diseases. Nat. Rev.. Cardiol..

[10] Agnew H.J., Kitson S.J., Crosbie E.J. (2023). Gynecological malignancies and obesity. Best Pract. Res. Clin. Obstet. Gynaecol..

[11] Maïano C., Hue O., Morin A.J., Moullec G. (2016). Prevalence of overweight and obesity among children and adolescents with intellectual disabilities: A systematic review and meta-analysis. Obes. Rev. Off. J. Int. Assoc. Study Obes..

[12] Kahathuduwa C.N., West B.D., Blume J., Dharavath N., Moustaid-Moussa N., Mastergeorge A. (2019). The risk of overweight and obesity in children with autism spectrum disorders: A systematic review and meta-analysis. Obes. Rev. Off. J. Int. Assoc. Study Obes..

[13] Mannan M., Mamun A., Doi S., Clavarino A. (2016). Prospective Associations between Depression and Obesity for Adolescent Males and Females- A Systematic Review and Meta-Analysis of Longitudinal Studies. PLoS ONE.

[14] Amiri S., Behnezhad S. (2019). Diabetes and anxiety symptoms: A systematic review and meta-analysis. Int. J. Psychiatry Med..

[15] Neggers Y.H., Goldenberg R.L., Ramey S.L., Cliver S.P. (2003). Maternal prepregnancy body mass index and psychomotor development in children. Acta Obstet. Gynecol. Scand..

[16] Hinkle S.N., Schieve L.A., Stein A.D., Swan D.W., Ramakrishnan U., Sharma A.J. (2012). Associations between maternal prepregnancy body mass index and child neurodevelopment at 2 years of age. Int. J. Obes..

[17] 1Basatemur E., Gardiner J., Williams C., Melhuish E., Barnes J., Sutcliffe A. (2013). Maternal prepregnancy BMI and child cognition: A longitudinal cohort study. Pediatrics.

[18] Gharipour M., Barekatain M., Sung J., Emami N., Sadeghian L., Dianatkhah M., Sarrafzadegan N., Jahanfar S. (2020). The Epigenetic Overlap between Obesity and Mood Disorders: A Systematic Review. Int. J. Mol. Sci..

[19] Flores-Dorantes M.T., Díaz-López Y.E., Gutiérrez-Aguilar R. (2020). Environment and Gene Association With Obesity and Their Impact on Neurodegenerative and Neurodevelopmental Diseases. Front. Neurosci..

[20] Nguyen J.C., Killcross A.S., Jenkins T.A. (2014). Obesity and cognitive decline: Role of inflammation and vascular changes. Front. Neurosci..

[21] American Psychiatric Association (2013). Diagnostic and Statistical Manual of Mental Disorders (DSM-5®).

[22] Bonti E., Giannoglou S., Georgitsi M., Sofologi M., Porfyri G.N., Mousioni A., Konsta A., Tatsiopoulou P., Kamari A., Vavetsi S. (2021). Clinical Profiles and Socio-Demographic Characteristics of Adults with Specific Learning Disorder in Northern Greece. Brain Sci..

[23] Wright B.A., Zecker S.G. (2004). Learning problems, delayed development, and puberty. Proc. Natl. Acad. Sci. USA.

[24] Snowling M.J., Gallagher A., Frith U. (2003). Family risk of dyslexia is continuous: Individual differences in the precursors of reading skill. Child Dev..

[25] Snowling M.J., Muter V., Carroll J. (2007). Children at family risk of dyslexia: A follow-up in early adolescence. J. Child Psychol. Psychiatry Allied Discip..

[26] Margai F., Henry N. (2003). A community-based assessment of learning disabilities using environmental and contextual risk factors. Soc. Sci. Med..

[27] McBride-Chang C., Lam F., Lam C., Chan B., Fong C.Y.C., Wong T.T.Y., Wong S.W. (2011). Early predictors of dyslexia in Chinese children: Familial history of dyslexia, language delay, and cognitive profiles. J. Child Psychol. Psychiatry.

[28] McBride-Chang C., Liu P.D., Wong T., Wong A., Shu H. (2012). Specific reading difficulties in Chinese, English, or both: Longitudinal markers of phonological awareness, morphological awareness, and RAN in Hong Kong Chinese children. J. Learn. Disabil..

[29] Goral M., Conner P.S. (2013). Language Disorders in Multilingual and Multicultural Populations. Annu. Rev. Appl. Linguist..

[30] Aruta S.F., Pruccoli J., Bandini N., Rucci P., Parmeggiani A. (2022). Specific Learning Disorders and Eating Disorders: An Italian retrospective study. Ital. J. Pediatr..

[31] Hay P., Mitchison D. (2019). Eating Disorders and Obesity: The Challenge for Our Times. Nutrients.

[32] Jebeile H., Lister N.B., Baur L.A., Garnett S.P., Paxton S.J. (2021). Eating disorder risk in adolescents with obesity. Obes. Rev. Off. J. Int. Assoc. Study Obes..

[33] Stabouli S., Erdine S., Suurorg L., Jankauskienė A., Lurbe E. (2021). Obesity and Eating Disorders in Children and Adolescents: The Bidirectional Link. Nutrients.

[34] Calcaterra V., Verduci E., Schneider L., Cena H., De Silvestri A., Vizzuso S., Vinci F., Mameli C., Zuccotti G. (2021). Sex-Specific Differences in the Relationship between Insulin Resistance and Adiposity Indexes in Children and Adolescents with Obesity. Children.

[35] Calcaterra V., Palombo C., Malacarne M., Pagani M., Federico G., Kozakova M., Zuccotti G., Lucini D. (2021). Interaction between Autonomic Regulation, Adiposity Indexes and Metabolic Profile in Children and Adolescents with Overweight and Obesity. Children.

[36] The WHO Child Growth Standards. https://www.who.int/tools/child-growth-standards/standards.

[37] Marshall W.A., Tanner J.M. (1969). Variations in pattern of pubertal changes in girls. Arch. Dis. Child..

[38] Marshall W.A., Tanner J.M. (1970). Variations in the pattern of pubertal changes in boys. Arch. Dis. Child..

[39] Matthews D.R., Hosker J.P., Rudenski A.S., Naylor B.A., Treacher D.F., Turner R.C. (1985). Homeostasis Model Assessment: Insulin Resistance and Beta-Cell Function from Fasting Plasma Glucose and Insulin Concentrations in Man. Diabetologia.

[40] Biswas A.B., Vahabzadeh A., Hobbs T., Healy J.M. (2010). Obesity in people with learning disabilities: Possible causes and reduction interventions. Nurs. Times.

[41] https://www.gov.uk/government/publications/obesity-weight-management-and-people-with-learning-disabilities/obesity-and-weight-management-for-people-with-learning-disabilities-guidance.

[42] Valerio G., Maffeis C., Saggese G., Ambruzzi M.A., Balsamo A., Bellone S., Bergamini M., Bernasconi S., Bona G., Calcaterra V. (2018). Diagnosis, treatment and prevention of pediatric obesity: Consensus position statement of the Italian Society for Pediatric Endocrinology and Diabetology and the Italian Society of Pediatrics. Ital. J. Pediatr..

[43] Görker I. (2019). The Prevalence and Gender Differences in Specific Learning Disorder. Learning Disabilities-Neurological Bases, Clinical Features and Strategies of Intervention.

[44] Veselinović A., Petrović S., Žikić V., Subotić M., Jakovljević V., Jeremić N., Vučić V. (2021). Neuroinflammation in Autism and Supplementation Based on Omega-3 Polyunsaturated Fatty Acids: A Narrative Review. Medicina.

[45] Moll K., Kunze S., Neuhoff N., Bruder J., Schulte-Körne G. (2014). Specific learning disorder: Prevalence and gender differences. PLoS ONE.

[46] Nass R.D. (1993). Sex differences in learning abilities and disabilities. Ann. Dyslexia.

[47] Ráčková L., Kuruczová D., Jarkovský J., Bienertová-Vašků J. (2021). Birth weight rather than birth length is associated with childhood behavioural problems in a Czech ELSPAC cohort. PLoS ONE.

[48] Suffren S., Angulo D., Ding Y., Reyes P., Marin J., Hernandez J.T. (2017). Long-term attention deficits combined with subcortical and cortical structural central nervous system alterations in young adults born small for gestational age. Early Hum. Dev..

[49] Isabelle G., Alexandre L., Sylvain R., Marie-Laure C., Jean-Christophe R., Stéphane M. (2011). Neurologic outcomes at school age in very preterm infants born with severe or mild growth restriction. Pediatrics.

[50] Squarza C., Picciolini O., Gardon L., Giannì M.L., Murru A., Gangi S., Cortinovis I., Milani S., Mosca F. (2016). Learning Disabilities in Extremely Low Birth Weight Children and Neurodevelopmental Profiles at Preschool Age. Front. Psychol..

[51] Jackson S.L., Cunningham S.A. (2015). Social Competence and Obesity in Elementary School. Am. J. Public Health.

[52] Lacagnina S. (2019). The Developmental Origins of Health and Disease (DOHaD). Am. J. Lifestyle Med..

[53] Buss C., Entringer S., Wadhwa P.D. (2012). Fetal programming of brain development: Intrauterine stress and susceptibility to psychopathology. Sci. Signal..

[54] Doi M., Usui N., Shimada S. (2022). Prenatal Environment and Neurodevelopmental Disorders. Front. Endocrinol..

[55] Seneviratne S.N., Rajindrajith S. (2022). Fetal programming of obesity and type 2 diabetes. World J. Diabetes.

[56] Azoulay L., Bouvattier C., Christin-Maitre S. (2022). Impact of intra-uterine life on future health. Ann. D’Endocrinol..

[57] Panera N., Mandato C., Crudele A., Bertrando S., Vajro P., Alisi A. (2022). Genetics, epigenetics and transgenerational transmission of obesity in children. Front. Endocrinol..

[58] Gaudieri P.A., Chen R., Greer T.F., Holmes C.S. (2008). Cognitive function in children with type 1 diabetes A Meta-Analysis. Diabetes Care.

[59] Maine A., Brown M., Truesdale M. (2020). Diabetes and people with learning disabilities: Issues for policy, practice, and education. Tizard Learn. Disabil. Rev..

[60] https://www.england.nhs.uk/rightcare/wp-content/uploads/sites/40/2017/11/rightcare-pathway-diabetes-reasonable-adjustments-learning-disability-2.pdf.

[61] Cacciatore M., Grasso E.A., Tripodi R., Chiarelli F. (2022). Impact of glucose metabolism on the developing brain. Front. Endocrinol..

